# *DOAP1* Promotes Flowering in the Orchid *Dendrobium* Chao Praya Smile

**DOI:** 10.3389/fpls.2017.00400

**Published:** 2017-03-23

**Authors:** Nunchanoke Sawettalake, Sumontip Bunnag, Yanwen Wang, Lisha Shen, Hao Yu

**Affiliations:** ^1^Department of Biology, Faculty of Science, Khon Kaen UniversityKhon Kaen, Thailand; ^2^Department of Biological Sciences and Temasek Life Sciences Laboratory, National University of SingaporeSingapore, Singapore

**Keywords:** *DOAP1*, MADS-box gene, flowering time, flower development, orchid, *Arabidopsis*

## Abstract

*APETALA1* (*AP1*) encodes a key MADS-box transcription factor that specifies the floral meristem identity on the flank of the inflorescence meristem, and determines the identity of perianth floral organs in the model plant *Arabidopsis thaliana*. Orchids are members of the Orchidaceae, one of the largest families of angiosperms. Although the expression patterns of a few *AP1*-like genes in orchids have been reported, their actual functions in orchid reproductive development are so far largely unknown. In this study, we isolated and characterized an *AP1* ortholog, *DOAP1*, from *Dendrobium* Chao Praya Smile. *DOAP1* was highly expressed in reproductive tissues, including inflorescence apices and flowers at various developmental stages. Overexpression of *DOAP1* resulted in early flowering in *Arabidopsis*, and was able to rescue the floral organ defects of *Arabidopsis ap1* mutants. Moreover, we successfully created transgenic *Dendrobium* Chao Praya Smile orchids overexpressing *DOAP1*, which displayed earlier flowering and earlier termination of inflorescence meristems into floral meristems than wild-type orchids. Our results demonstrate that *DOAP1* plays an evolutionarily conserved role in promoting flowering and floral meristem specification in the Orchidaceae family.

## Introduction

During the floral transition in flowering plants, the vegetative shoot apical meristem that produces leaves is transformed into the inflorescence meristem that produces flowers. This process is controlled by flowering regulatory networks in response to various environmental and endogenous signals, and the underlying molecular mechanisms have been intensively studied in the model plant *Arabidopsis thaliana*. In *Arabidopsis*, environmental and endogenous flowering signals perceived by various genetic pathways mediate transcriptional regulation of two major floral pathway integrators, *FLOWERING LOCUS T* (*FT*) and *SUPPRESSOR OF OVEREXPRESSION OF CONSTANS 1* (*SOC1*), which in turn activate two floral meristem identity genes, *APETALA1* (*AP1*) and *LEAFY* (*LFY*), to regulate the specification and formation of floral meristems on the flank of the shoot apical meristem (Kardailsky et al., 1999; Kobayashi et al., 1999; Blazquez and Weigel, 2000; Lee et al., 2000; Samach et al., 2000; Liu et al., 2009).

*AP1* encodes a MADS-box transcription factor that not only specifies the floral meristem identity, but also contributes to determination of the identity of perianth floral organs in *Arabidopsis* (Irish and Sussex, 1990; Mandel et al., 1992; Bowman et al., 1993). When the activity of *AP1* is lost, floral meristems that would normally develop into flowers are partially converted into inflorescence meristems. In *ap1* mutants, flowers exhibited transformation of sepals into leaf-like organs and failure of petal development. In contrast, overexpression of *AP1* results in early flowering and transformation of inflorescence meristems into floral meristems (Mandel and Yanofsky, 1995). During the floral transition, *AP1* expression is directly activated by LFY and a protein complex consisting of FT and FD (Wagner et al., 1999; Abe et al., 2005; Wigge et al., 2005). In emerging floral meristems, AP1 plays dual functions as an activator and a repressor. It activates B class homeotic genes to mediate the specification of petals (Hill et al., 1998; Ng and Yanofsky, 2001), while it also suppresses a group of flowering time genes to prevent the reversion of floral meristems into inflorescence meristems (Yu et al., 2004; Liu et al., 2007; Kaufmann et al., 2010). Moreover, AP1 directly regulates the homeostasis of cytokinin by suppressing its biosynthesis and activating its degradation to establish determinate floral meristems (Han et al., 2014).

So far *AP1* orthologs have been isolated in a wide range of plant species, such as pea *(Pisum sativum)* (Berbel et al., 2001), apple *(Malus domestica)* (Kotoda et al., 2002), common wheat (*Triticum aestivum*) (Adam et al., 2007), moth orchid (*Phalaenopsis* ‘Hatsuyuki’) (Song et al., 2011), longan (*Dimocarpus longan)* (Winterhagen et al., 2013), trifoliate orange (*Poncirus trifoliata* L. Raf.) (Sun et al., 2014), Birch (*Betula platyphylla*) (Huang et al., 2014) and poplar (*Populus tomentosa*) (Chen et al., 2015). Through mainly examining their expression patterns and their effects on heterologous systems, most of these genes have been suggested to affect either the flowering process or floral organ formation.

*Dendrobium* is one of the largest genera in the family Orchidaceae, which is one of the largest families of angiosperms (Teixeira da Silva et al., 2014). The *Dendrobium* genus comprises more than 1200 species, and its members have attractive ornamental and medicinal values (Yu and Goh, 2001; Teixeira da Silva et al., 2014). The high demand of *Dendrobium* orchids has enabled *Dendrobium* growers to create many varieties and hybrids with various floral traits. However, the long vegetative phase and low survival rate of orchid seedlings under natural environmental conditions make it difficult to propagate and breed *Dendrobium* orchids (Yu and Goh, 2001; Chai and Yu, 2007; Wang et al., 2009). Therefore, it is important to elucidate the molecular mechanisms underlying the floral transition in orchids so as to identify appropriate regulators for targeted manipulation or screening of orchid traits. Recent studies have isolated and characterized several orthologs of *Arabidopsis* floral pathway integrators in orchids. Overexpression of three orthologs of *FT*, *OnFT*, *DnFT* and *CgFT* from *Oncidium*, *Dendrobium*, and *Cymbidium* orchids, respectively, causes early flowering in transgenic *Arabidopsis* or tobacco plants (Hou and Yang, 2009; Li et al., 2012; Xiang et al., 2012). In addition, overexpression of *DOSOC1*, an ortholog of the *Arabidopsis SOC1*, promotes flowering in both transgenic *Arabidopsis* and *Dendrobium* orchids (Ding et al., 2013).

In this study, we isolated an *AP1* ortholog, *DOAP1*, from *Dendrobium* Chao Praya Smile, and characterized its function through examining its expression patterns and investigating its biological effects in both transgenic *Arabidopsis* and orchid plants. Our results suggest that *DOAP1* may play a conserved role in promoting flowering and floral meristem development in the Orchidaceae family.

## Materials and Methods

### Plant Material and Growth Conditions

*Dendrobium* Chao Praya Smile (a hybrid of *Dendrobium* Pinky and *Dendrobium* Kiyomi Beauty) plants were grown in pots in the greenhouse at 28 ± 4°C under natural lighting conditions. In our *in vitro* culture system for *Dendrobium* Chao Praya Smile, protocorms developing from seeds were used as the starting materials. They were cultured in modified liquid Knudson C (KC) medium supplemented with 2% (w/v) sucrose, 15% (v/v) coconut water and 4.4 μM benzyladenine (BA) at 24°C under a 16-h photoperiod on rotary shakers at 120 rpm (Yu and Goh, 2000a; Ding et al., 2013). Plantlets at the floral transitional stage were transferred to the two-layer modified KC medium as previously described (Hee et al., 2007; Sim et al., 2007). Wild-type *Arabidopsis thaliana* ecotype Columbia (Col-0) and *35S:DOAP1* transgenic plants in both wild-type and *ap1-10* Col-0 backgrounds were grown under long-day conditions (16 h light/8 h dark) at 23 ± 2°C.

### Isolation of *DOAP1*

Total RNA was extracted from inflorescences of *Dendrobium* Chao Praya Smile using the RNeasy^®^ Plant Mini Kit (QIAGEN). To isolate the putative *AP1* orthologs, two degenerate primers, 5′-CAGCTGARGCGRATMGAGAAC-3′ and 5′-GCKMAGCATCCAWGGYGG-3′, were manually designed based on the conserved regions among *AP1* orthologs in various plant species. Based on the partial cDNA sequence obtained, the full-length cDNA of *DOAP1* was further amplified using the SMARTER^TM^ RACE cDNA Amplification Kit (BD Biosciences Clontech).

### Sequence Analysis

Protein sequences of *AP1*-like genes were retrieved from the National Center for Biotechnology Information (NCBI) database. Alignment of amino acid sequences was performed using the Clustal Omega multiple sequence alignment program^1^ and BOXSHADE 3.21^2^. The phylogenetic tree was generated by the neighbor-joining algorithm using the MEGA6 software^3^.

### Expression Analysis

Total RNA was extracted from orchids or *Arabidopsis* plants using the RNeasy^®^ Plant Mini Kit (QIAGEN), and reverse-transcribed using the SuperScript^TM^ II Reverse Transcriptase Kit (Invitrogen) according to the manufacturer’s instructions. Real-time PCR reaction was performed in triplicates on the CFX384 Real-Time PCR Detection System (Bio-Rad) with the SYBR Green Master Mix (Toyobo). *UBIQUITIN* (*DOUbi*) in *Dendrobium* Chao Praya Smile was used as a reference gene (Ding et al., 2013). Calculation of relative gene expression levels was performed as previously reported (Liu et al., 2007). Semi-quantitative reverse transcription PCR (RT-PCR) was carried out as previously reported (Xu et al., 2006) using either *Arabidopsis β-TUBLIN2* (*TUB2*) or orchid *ACTIN* (*DOActin*) as a reference gene.

### *Arabidopsis* Transformation

The full-length *DOAP1* cDNA fragment was cloned into pGreen0229-35S under the control of two CaMV *35S* promoters (Yu et al., 2004). The resulting construct was introduced into *Agrobacterium tumefaciens* GV3101 competent cells by electroporation. *35S:DOAP1* transgenic *Arabidopsis* lines were created by *Agrobacterium tumefaciens*-mediated transformation and selected by 0.3 g/L Basta on soil.

### Orchid Transformation by *Agrobacterium*-Mediated Transformation

Genetic transformation of *Dendrobium* Chao Praya Smile was performed according to the reported L-methionine sulfoximine (MSO) selection system with minor modifications (Yu et al., 2001; Chai et al., 2007; Ding et al., 2013). *Dendrobium* Chao Praya Smile calli were cut into small pieces 3–5 mm in diameter and then cultured in modified KC liquid medium supplemented with 2% (w/v) sucrose, 15% (v/v) coconut water and 4.4 μM BA. *Agrobacterium* pellet was re-suspended in KC liquid medium to be co-cultivated with prepared orchid calli. Acetosyringone (100 μM) was added to induce transformation. After co-cultivation for 2 h, calli were placed on solid KC medium in the dark for three nights. *Agrobacterium*-infected calli were rinsed by water containing 200 mg/L cefotaxime to remove the bacteria, and grown on solid KC medium containing 0.5 μM MSO as a selection agent. Surviving calli were subcultured onto fresh solid medium every 20 days. After four rounds of MSO selection, green calli were transferred to modified KC solid medium containing 2 μM MSO for lethal selection. After three rounds of selection, putative surviving transgenic lines were cultured on modified KC solid medium for further investigation.

### Southern Blot

Genomic DNA isolated from wild-type and *35S:DOAP1* transgenic orchids was digested by *Eco*RI for 16 h, resolved on 1.2% (w/v) agarose gels, and then transferred onto nylon membranes. Blots were hybridized overnight with the specific digoxigenin-labeled DNA, washed and detected in accordance with the manufacturer’s instructions (DIG Application Manual for Filter Hybridization, Roche) as previously reported (Yu and Goh, 2000b).

## Results

### Isolation of *DOAP1* from *Dendrobium* Chao Praya Smile

To isolate *AP1*-like genes from *Dendrobium* Chao Praya Smile, we designed a pair of degenerate primers based on the conserved amino acid sequences of *AP1* orthologs in various plant species. A partial cDNA fragment was obtained by reverse transcription PCR using RNA extracted from inflorescence apices of *Dendrobium* Chao Praya Smile. Since this fragment showed high sequence similarity with other *AP1*-like genes, we further designed primers based on this fragment and obtained the corresponding full-length cDNA sequence, designated *DOAP1* (GenBank accession No. KY471451), using the rapid amplification of cDNA ends (RACE) method.

**FIGURE 1:**
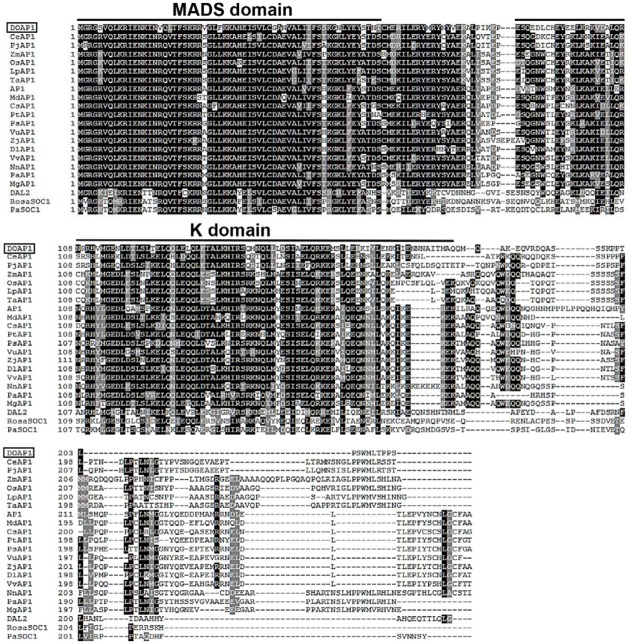
**Alignment of the amino acid sequences of DOAP1 and its orthologs from other plant species**. Conserved residues are shown in black, while similar residues are shown in gray. Dashes indicate gaps to maximize alignment. The conserved MADS and K domains are indicated. The species names are shown behind their corresponding protein names as follows: DOAP1, *Dendrobium* Chao Praya Smile; CeAP1, *Cymbidium ensifolium*; PjAP1: *Phalaenopsis japonica*; ZmAP1, *Zea mays*; OsAP1, *Oryza sativa*; LpAP1, *Lolium perenne*; TaAP1, *Triticum aestivum*; AP1, *Arabidopsis thaliana*; MdAP1, *Malus domestica*; CsAP1, *Citrus sinensis*; PtAP1, *Populus trichocarpa*; PsAP1, *Paeonia suffruticosa*; VuAP1, *Vigna unguiculata*; ZjAP1, *Ziziphus jujube*; DlAP1, *Dimocarpus longan*; VvAP1, *Vitis vinifera*; NnAP1, *Nelumbo nucifera*; PaAP1, *Persea Americana*; MgAP1, *Magnolia grandiflora*. Non-AP1-like proteins that are encoded by other MADS-box subfamily genes were used as outlier in this alignment as follows: DEFICIENS-AGAMOUS-LIKE2 (DAL2) of *Picea abies*, and two SUPPRESSOR OF OVEREXPRESSION OF CONSTANS 1 (SOC1)-like proteins from *Rosa* hybrid cultivar (RosaSOC1) and *Picea abies* (PaSOC1).

*DOAP1* cDNA is 897 bp in length with a 639 bp coding region. The deduced DOAP1 amino acid sequence contains a highly conserved MADS domain and a less conserved K domain, and a diverse C terminal region as found in other *AP1*-like genes from various plant species (**Figure 1**). Multiple sequence alignment showed that DOAP1 shared the highest sequence identity with other orchid AP1 orthologs, such as CeAP1 (*Cymbidium ensifolium*; 69.95% identity) and PjAP1 (*Phalaenopsis japonica*; 62.56% identity). DOAP1 also had higher sequence identity with monocot AP1 orthologs, such as TaAP1 (*Triticum aestivum*; 58% identity), OsAP1 (*Oryza sativa*; 56.72% identity) and ZmAP1 (*Zea mays*; 54.59% identity), than *Arabidopsis* AP1 (53.3% identity).

**FIGURE 2:**
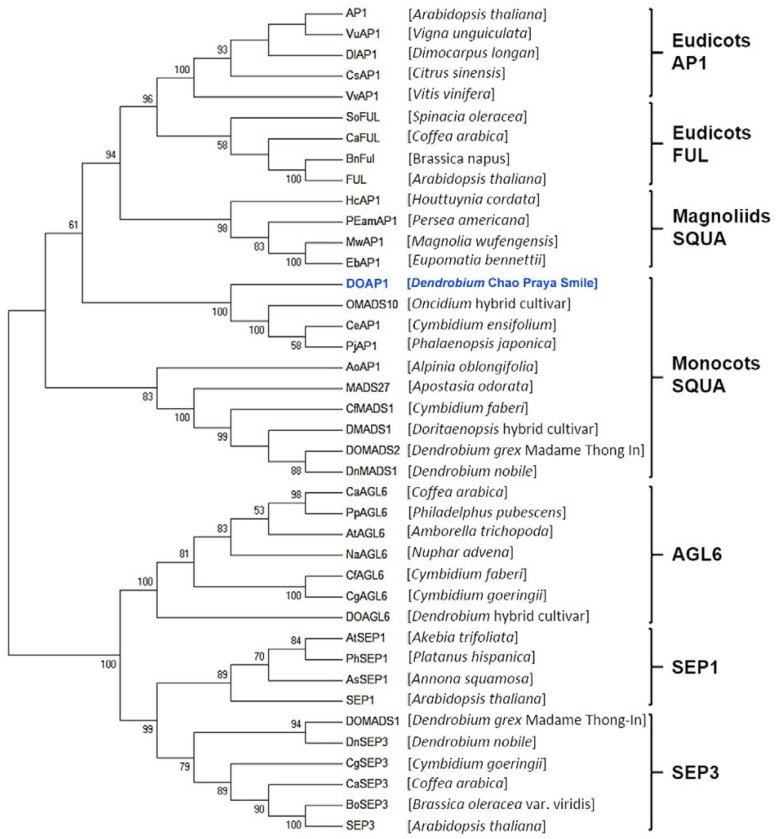
**Phylogenetic tree of AP1/AGL9 -like MADS-box proteins**. Amino acid sequences of AP1/AGL9 –like MADS box genes were obtained from the NCBI database. DOAP1 investigated in this study is highlighted in blue. The multiple sequence alignment was performed via Clustal Omega. The tree was constructed with MEGA6 using the neighbor-joining method. Genus names of respective species are given in the parentheses behind the corresponding protein names. Bootstrap values (>50%) in 1,000 replicates are shown below the nodes.

To determine the evolutionary relationship between DOAP1 and other AP1-like proteins from other plant species, we constructed a phylogenetic tree based on the analysis of amino acid sequences of MIK regions (**Figure 2**). The tree showed that DOAP1 was clustered together with other orchid AP1-like proteins, such as OMADS10 (Chang et al., 2009) and DOMADS2 (Yu and Goh, 2000b), in the monocotyledonous subgroup of SQUA that includes AP1-like proteins isolated from monocots (Purugganan et al., 1995; Theissen et al., 1996).

### *DOAP1* Expression Pattern in *Dendrobium* Chao Praya Smile

**FIGURE 3:**
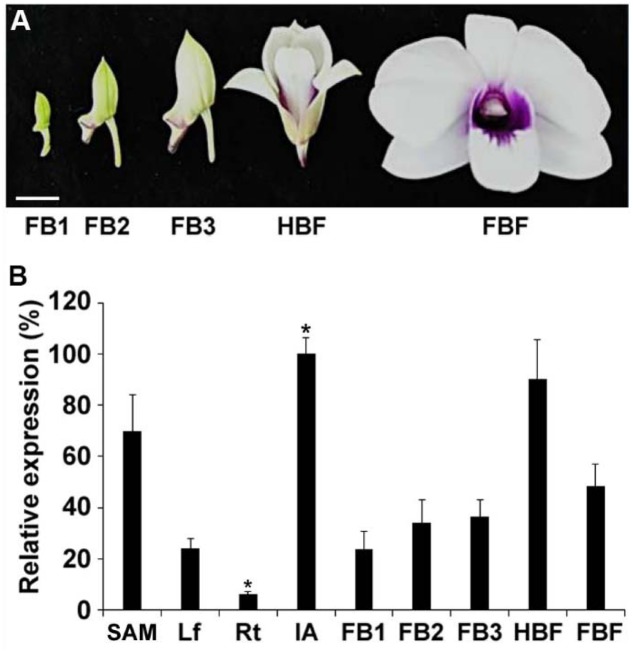
**Expression of *DOAP1* in various tissues of *Dendrobium* Chao Praya Smile**. **(A)** Five developmental stages of flowers, including floral bud 1 (FB1), FB2, FB3, half-bloomed flower (HBF), and fully-bloomed flower (FBF), are shown. Scale bar, 1 cm. **(B)** Quantitative analysis of *DOAP1* expression in various tissues and different floral developmental stages of *Dendrobium* Chao Praya Smile. Transcript levels were determined by quantitative real-time PCR analyses of three independently harvested samples. The levels of gene expression normalized to the orchid polyubiquitin gene (*DOUbi*) expression are shown relative to the highest level set at 100%. SAM, vegetative shoot apical meristem; Lf, leaf; Rt, root; IA, inflorescence apex; FB, floral bud; HBF, half-bloomed flower; FBF, fully bloomed flower. Error bars, mean ± SD. Asterisks indicate statistically significant differences in the expression level of *DOAP1* in IA or Rt as compared to its expression levels in any other tissues examined (two-tailed paired Student’s *t*-test, *p* < 0.05).

To study the expression pattern of *DOAP1* in *Dendrobium* Chao Praya Smile, cDNA was synthesized from RNA extracted from various orchid tissues, including leaves, roots, inflorescence apices, floral buds at different stages (stages 1–3, at which buds were 0.5–1 cm, 1–2 cm, and over 2 cm in length, respectively), half-bloomed flowers and fully bloomed flowers (**Figure 3A**). Quantitative real-time PCR analysis showed that *DOAP1* transcripts were detected at the highest level in inflorescence apices and at the lowest level in roots (**Figure 3B**). Notably, *DOAP1* expression was significantly higher in inflorescence apices than in vegetative shoot apices. Moreover, *DOAP1* expression gradually increased in developing floral buds at various stages and half-bloomed flowers, but decreased afterward in fully bloomed flowers (**Figure 3B**). These expression patterns indicate that *DOAP1* function may be closely associated with flowering time control and flower development in *Dendrobium* Chao Praya Smile.

### *DOAP1* Promotes Flowering and Partially Complements *ap1* Mutants in *Arabidopsis*

**FIGURE 4:**
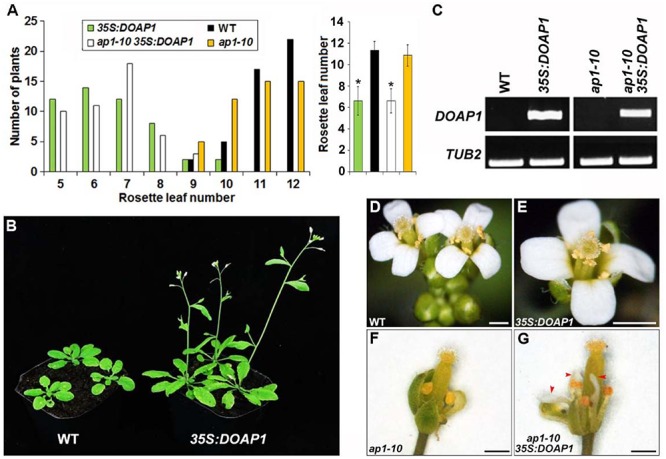
**Overexpression of *DOAP1* in *Arabidopsis* results in early flowering**. **(A)** Flowering time of *35S:DOAP1* in wild-type and *ap1-10* backgrounds. The left panel shows the distribution of flowering time in T1 transgenic lines harboring *35S:DOAP1* in wild-type and *ap1-10* backgrounds grown under long day conditions, while the right panel shows the average flowering time of various transgenic lines. Error bars, mean ± SD. Asterisks indicate statistically significantly differences in flowering time of *35S:DOAP1* and *ap1-10 35S:DOAP1* compared to wild-type and *ap1-10*, respectively (two-tailed paired Student’s *t*-test, *p* < 0.05). **(B)** Progenies of a representative *35S:DOAP1* line (right) flower earlier than wild-type plants (left) under long day conditions. **(C)** Examination of *DOAP1* expression in representative *35S:DOAP1* and *ap1-10 35S:DOAP1* lines by semi-quantitative RT-PCR. *TUB2* was amplified as an internal control. **(D–G)** Floral phenotypes of *35S:DOAP1* and *ap1-10 35S:DOAP1*. There is no visible difference in floral morphology of wild-type **(D)** and *35S:DOAP1* **(E)** plants, whereas deficiency in petal formation in *ap1-10* **(F)** was partially rescued in *ap1 35S:DOAP1* **(G)**. Red arrowheads indicate petals in *ap1 35S:DOAP1*. Bars represent 5 mm.

In order to investigate the biological function of *DOAP1*, we firstly generated transgenic *Arabidopsis* plants harboring the *DOAP1* coding sequence driven by the CaMV *35S* promoter. Among 50 independent *35S:DOAP1* transgenic lines created at the T1 generation, 46 lines showed an early flowering phenotype typically producing only 5–7 rosette leaves as compared to wild-type plants, which developed 9–12 rosette leaves under long day conditions (**Figures 4A,B**). Semi-quantitative RT-PCR using *DOAP1*-specific primers confirmed that *DOAP1* was overexpressed in a representative *35S:DOAP1* transgenic *Arabidopsis* line with a single T-DNA insertion (**Figure 4C**), implying that the early flowering phenotype of *35S:DOAP1* is associated with overexpression of *DOAP1*. In addition to the defect in flowering time, the inflorescence meristems of *35S:DOAP1* transgenic lines usually terminated as flowers after producing only a few flowers, while these flowers produced floral organs indistinguishable from those of wild-type flowers (**Figures 4D,E**), suggesting that overexpression of *DOAP1* promotes the formation of floral meristems, but does not affect floral organ identity in *Arabidopsis*.

We further performed *Agrobacterium*-mediated transformation to introduce *35S:DOAP1* into *ap1-10* mutants, which were generated from ethyl methanesulfonate-mutagenized populations of *Arabidopsis* ecotype Columbia (Schultz and Haughn, 1993), to examine whether *DOAP1* could complement the loss function of *AP1*. While *ap1-10* exhibited comparable flowering time to wild-type plants, most of the *ap1-10 35S:DOAP1* lines flowered much earlier than wild-type and *ap1-10* plants, a flowering pattern resembling *35S:DOAP1* in the wild-type background (**Figure 4A**). We further identified several *ap1-10 35S:DOAP1* transgenic lines with single T-DNA insertion, which consistently displayed the similar early flowering phenotype at various generations, and selected one representative line for further investigations. Like other *ap1* mutants, *ap1-10* showed typical defects in the identity of perianth organs, such as homeotic transformation of petals into stamens or stamen-petal chimeric structures (**Figure 4F**). In contrast, petal formation was restored in *ap1-10 35S:DOAP1* (**Figure 4G**), demonstrating that DOAP1 plays the same role as AP1 in regulating petal formation in *Arabidopsis*. Similarly, semi-quantitative RT-PCR detected high expression of *DOAP1* in the representative *ap1-10 35S:DOAP1* line (**Figure 4C**), substantiating a causal link between the observable phenotypes and overexpression of *DOAP1* in *ap1-10 35S:DOAP1*.

### *DOAP1* Promotes Early Formation of Inflorescences in *Dendrobium* Chao Praya Smile

**FIGURE 5:**
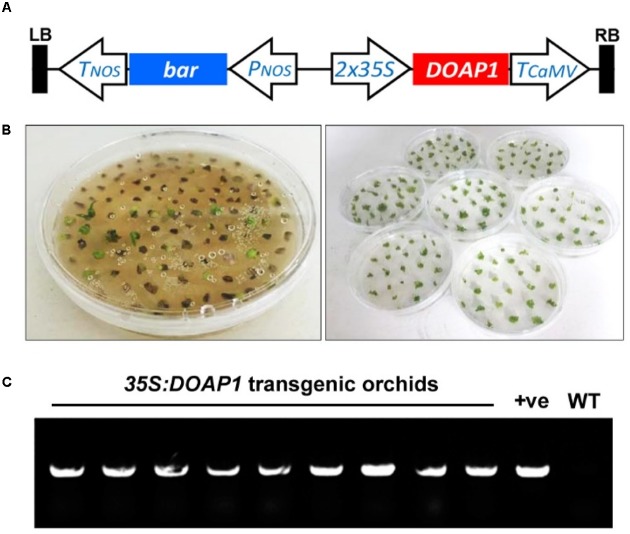
**Generation of *35S:DOAP1* transgenic orchids**. **(A)** Schematic diagram of the T-DNA region of the *pGreen0229-35S:DOAP1* construct. LB, left border; Tnos, nopaline synthase terminator sequence; *bar*, bialaphos resistance gene; Pnos, nopaline synthase promoter sequence; *35S*, Cauliflower mosaic virus *35S* promoter sequence; TCaMV, Cauliflower mosaic virus terminator sequence; RB, right border. **(B)** Genetic transformation of *Dendrobium* Chao Praya Smile using the MSO selection system. After lethal section on the solid medium containing 2 μM MSO (left panel), green surviving calli were further subcultured onto fresh medium every 20 days (right panel). **(C)** PCR genotyping of *DOAP1* transgenic orchids shows the presence of *35S:DOAP1* transgene in nine independent lines of *Dendrobium* Chao Praya Smile. The specific primers from the *35S* promoter and *DOAP1*, respectively, were designed for genotyping. The *35S:DOAP1* plasmid and the genomic DNA of wild-type orchids were used as a positive control (+ve) and a negative control (WT), respectively.

To understand the endogenous function of *DOAP1* in *Dendrobium* Chao Praya Smile, we created transgenic orchids bearing *35S:DOAP1* in a pGreen vector (**Figure 5A**) using an integrated orchid gene transformation and *in vitro* orchid culture system (Yu et al., 2001; Chai et al., 2007; Ding et al., 2013).

After *Agrobacterium*-mediated transformation of *Dendrobium* Chao Praya Smile calli, the transformed materials were selected on the medium containing 0.5 and 2 μM MSO as a selection agent for initial and lethal selection (**Figure 5B**), respectively. After a total of seven rounds of selection, most of the calli turned necrotic and eventually died, while some putative transformants survived and proliferated into protocorm-like bodies, which further developed into young plantlets. We screened out nine independent *35S:DOAP1* transgenic *Dendrobium* Chao Praya Smile lines, and confirmed the presence of the *35S:DOAP1* transgene in these lines by PCR genotyping using the specific primers from *35S* and *DOAP1* (**Figure 5C**).

**FIGURE 6:**
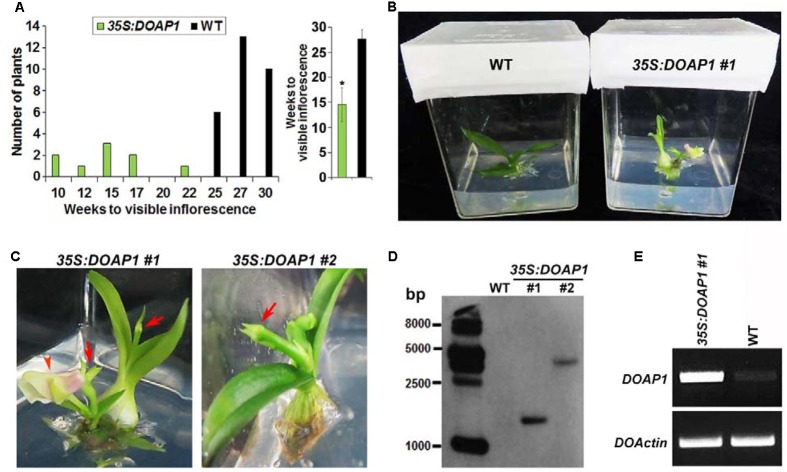
**Overexpression *DOAP1* in *Dendrobium* Chao Praya Smile causes early flowering**. **(A)** Comparison of flowering time of wild-type and *35S:DOAP1* transgenic orchids. The left panel shows that all 9 transgenic lines produce inflorescences (indicated by the visible inflorescence stalk) earlier than 29 wild-type plants, while the right panel shows the comparison of the average time to produce visible inflorescences of *35S:DOAP1* and wild-type orchids. Error bars, mean ± SD. Asterisk indicates a statistically significant difference in flowering time between *35S:DOAP1* and wild-type (two-tailed paired Student’s *t*-test, *p* < 0.05). **(B)** One representative *35S:DOAP1* transgenic orchid (#1) shows earlier flowering than a wild-type (WT) orchid at 16 weeks after *in vitro* culture under our growth conditions. **(C)** Close views of two independent *35S:DOAP1* transgenic lines (#1 and #2). Arrows indicate inflorescence apices, while arrowhead indicates a flower. **(D)** Southern blot analysis of the presence of the *35S:DOAP1* transgene in transgenic orchids. The genomic DNA extracted from wild-type and two *35S:DOAP1* transgenic lines (#1 and #2) was digested with *Eco*RI, and the DNA gel blot was hybridized with the digoxigenin-labeled probe that was synthesized from the *bar* gene. The sizes of the DNA markers are given on the left in bases. **(E)** Examination of *DOAP1* expression in wild-type and *35S:DOAP1* (#1) orchids by semi-quantitative RT-PCR. Total RNA was extracted from inflorescence apices, and *DOActin* was amplified as an internal control.

We then compared the growth status of wild-type and *35S:DOAP1 Dendrobium* Chao Praya Smile plants using our established *in vitro* culture system (Ding et al., 2013), which allows rapid *in vitro* development of orchid plants from the vegetative to reproductive phase. Under our growth conditions, it took wild-type orchid plants 25–30 weeks to grow from protocorm-like bodies to plantlets with the first visible inflorescence stalks (**Figure 6A**), whereas 8 out of 9 *35S:DOAP1* transgenic lines displayed the first visible inflorescences at 10–17 weeks of culture (**Figures 6A,B**). These phenotypes indicate a role of *DOAP1* in promoting the transition of vegetative shoot apical meristems into inflorescence meristems. As compared to wild-type inflorescences (Ding et al., 2013), the inflorescence apices of these *35S:DOAP1* transgenic plants were usually terminated as floral buds immediately after producing only 1–2 floral buds or even without generating any other floral structure (**Figure 6C**), suggesting a quick transformation of inflorescence meristems into floral meristems in *35S:DOAP1*. In addition, Southern blot hybridization revealed the presence of the T-DNA region containing the bialaphos resistance (*bar*) gene in the genome of *35S:DOAP1* #1 and #2 lines (**Figure 6D**), which substantiated the results showing the presence of the *35S:DOAP1* transgene in the *35S:DOAP1* genome (**Figure 5C**). As expected, semi-quantitative RT PCR showed that *DOAP1* was overexpressed in inflorescence apices of *35S:DOAP1* transgenic orchids compared to wild-type orchids (**Figure 6E**). These results imply that the phenotypes of *35S:DOAP1* transgenic orchids are associated with overexpression of *DOAP1* in these plants.

## Discussion

Although the Orchidaceae is one of the largest and most widespread families of flowering plants, the molecular mechanism underlying the flowering process of the members in this family remain largely unknown. The difficulty in performing molecular genetic studies on orchids is partially due to their long vegetative phase and recalcitrant nature for gene transformation. In this study, we have taken advantage of an established *Agrobacterium*-mediated gene transformation system based on MSO selection coupled with *in vitro* orchid culture system (Yu et al., 2001; Hee et al., 2007; Sim et al., 2007; Ding et al., 2013) to study the biological function of a newly isolated *AP1* ortholog, *DOAP1*, from *Dendrobium* Chao Praya Smile.

Our findings have provided several pieces of evidence to support that *DOAP1* plays a conserved role in promoting the floral transition and specifying the identity of perianth floral organs. First, in addition to the conserved MADS and K domains found among AP1-like proteins, phylogenetic analysis has revealed that DOAP1 and other orchid AP1-like proteins are assigned to the same clade in the monocotyledonous subgroup of SQUA, which contains AP1/SQUA-like proteins from monocots (Purugganan et al., 1995; Theissen et al., 1996). Second, *DOAP1* is highly expressed in inflorescence apices and flowers at various developmental stages, which is similar to the patterns exhibited by most of *AP1*-like genes studied (Becker and Theissen, 2003; Litt and Irish, 2003). Notably, *DOAP1* is also expressed in vegetative shoot apices and upregulated in inflorescence apices, suggesting that it could be one of the earliest regulatory genes that promote the floral transition in orchids. Third, overexpression of *DOAP1* in both *Arabidopsis* and orchids causes early flowering and early termination of inflorescence meristems as floral meristems, which are similar to the effects of overexpression of *AP1* in *Arabidopsis* (Mandel and Yanofsky, 1995), indicating that like *AP1*, *DOAP1* may serve as a floral meristem identity gene to promote the specification of floral meristems in both *Arabidopsis* and orchids. Similarly, overexpression of *AP1* orthologs from other plant species also causes early flowering in transgenic plants (Berbel et al., 2001; Kotoda et al., 2002; Adam et al., 2007; Huang et al., 2014; Sun et al., 2014), suggesting a conserved role of *AP1*-like genes in accelerating flowering probably through promoting the early formation of floral meristems. Last, overexpression of *DOAP1* in *ap1-10 Arabidopsis* mutants partially rescues the defect in petal formation, suggesting that *DOAP1* and *AP1* also share a similar role as class A organ identity genes (Irish and Sussex, 1990; Mandel et al., 1992; Bowman et al., 1993). Taken together, our findings suggest that *DOAP1* may play evolutionarily conserved roles in promoting floral meristem formation and specification of perianth organs in the Orchidaceae family. As AP1 has been shown to prevent the formation of flowers in the axils of sepals by directly regulating the cytokinin homeostasis (Han et al., 2014), it would be interesting to investigate whether DOAP1 also has a similar role in orchids in future studies.

Despite the functional conservation of *DOAP1* as discussed above, some of our results also indicate other potential functions of *DOAP1* in orchid development. For example, *DOAP1* is expressed in both leaves and floral buds at stage 1 almost at comparable levels (**Figure 3B**), implying its potential involvement in vegetative growth. In addition, there is a dramatic decrease in *DOAP1* expression in fully bloomed flowers as compared to its expression in half-bloomed flowers (**Figure 3B**). This change could be relevant to a possible role of *DOAP1* in late flower development. Consistently, several other orchid *AP1*-like genes also exhibit potential functions in various aspects of flower development other than their conserved roles in floral meristem development and perianth organ specification. For example, another orchid *AP1*-like gene, *DOMADS2*, isolated from *Dendrobium* Madame Thong-In is not only highly expressed in the shoot apical meristem during the floral transition, but also expressed in orchid reproductive organs, such as column (gynostemium, a fused structure of stigmas, styles and stamens) and ovary (Yu and Goh, 2000b), indicating that *DOMADS2* may also be involved in reproductive organ development. Similarly, another *AP1*-like gene, *EpMADS12*, in *Erycina pusilla* is also detectable in multiple floral organs, such as lips, column, and pollinarium (Lin et al., 2016). These diverse expression patterns of *AP1*-like genes indicate that *AP1*-like genes may evolve with multiple functions in regulating flowering and flower development in orchids. Further investigation of these orchid *AP1*-like genes through knocking down or knocking out their expression in orchids will shed light on their endogenous functions in orchid reproductive development.

## Author Contributions

NS, YW, LS, and HY conceived and designed the study. NS and SB performed the experiments. NS, SB, YW, LS, and HY analyzed data. NS, YW, LS, and HY wrote the paper. All authors read and approved the manuscript.

## Conflict of Interest Statement

The authors declare that the research was conducted in the absence of any commercial or financial relationships that could be construed as a potential conflict of interest.

## Footnotes

1 http://www.ebi.ac.uk/Tools/msa/clustalo/

2 http://www.ch.embnet.org/software/BOX_form.html

3 http://www.megasoftware.net/
